# The diversity of tick-borne bacteria and parasites in ticks collected from the Strandja Nature Park in south-eastern Bulgaria

**DOI:** 10.1186/s13071-018-2721-z

**Published:** 2018-03-20

**Authors:** Julian Nader, Nina Król, Martin Pfeffer, Valerie Ohlendorf, Marco Marklewitz, Christian Drosten, Sandra Junglen, Anna Obiegala

**Affiliations:** 1 Institute of Animal Hygiene and Veterinary Public Health, University of Leipzig, Leipzig, Germany; 2 Institute of Virology, Charité - Universitätsmedizin Berlin, Free University Berlin, Humboldt-University Berlin, and Berlin Institute of Health, Berlin, Germany; 3 German Center for Infection Research (DZIF), Berlin, Germany

**Keywords:** *Ixodes ricinus*, *Dermacentor reticulatus*, *Rhipicephalus*, *Rickettsia*, *Anaplasma phagocytophilum*, *Borrelia*, *Babesia*, “*Candidatus* Neoehrlichia mikurensis”, Bulgaria

## Abstract

**Background:**

Ticks are important carriers of many different zoonotic pathogens. To date, there are many studies about ticks and tick-borne pathogens (TBP), but only a few were carried out in Bulgaria. The present study intends to detect the prevalence of tick-borne bacteria and parasites occurring at the Black Sea in Bulgaria to evaluate the zoonotic potential of the tick-borne pathogens transmitted by ticks in this area.

**Methods:**

In total, cDNA from 1541 ticks (*Dermacentor* spp., *Haemaphysalis* spp., *Hyalomma* spp., *Ixodes* spp. and *Rhipicephalus* spp.) collected in Bulgaria by flagging method or from hosts was tested in pools of ten individuals each for *Anaplasma phagocytophilum*, *Babesia* spp., *Borrelia burgdorferi* (*s*.*l*.), *Rickettsia* spp. and “*Candidatus* Neoehrlichia mikurensis” *via* conventional and quantitative real-time PCR. Subsequently, samples from positive pools were tested individually and a randomized selection of positive PCR samples was purified, sequenced, and analyzed.

**Results:**

Altogether, 23.2% of ticks were infected with at least one of the tested pathogens. The highest infection levels were noted in nymphs (32.3%) and females (27.5%). Very high prevalence was detected for *Rickettsia* spp. (48.3%), followed by *A. phagocytophilum* (6.2%), *Borrelia burgdorferi* (*s*.*l*.) (1.7%), *Babesia* spp. (0.4%) and “*Ca*. Neoehrlichia mikurensis” (0.1%). Co-infections were found in 2.5% of the tested ticks (mainly *Ixodes* spp.). Sequencing revealed the presence of *Rickettsia monacensis*, *R. helvetica*, and *R. aeschlimannii*, *Babesia microti* and *B. caballi*, and *Theileria buffeli* and *Borrelia afzelli.*

**Conclusion:**

This study shows very high prevalence of zoonotic *Rickettsia* spp. in ticks from Bulgaria and moderate to low prevalence for all other pathogens tested. One should take into account that tick bites from this area could lead to *Rickettsia* infection in humans and mammals.

## Background

It is well known that ticks are distributed all over the world and may transmit zoonotic diseases. The majority of studies on ticks and tick-borne diseases (TBD) in Europe are focused on central, southern and eastern Europe. Bulgarian studies on this matter are scarce. Little is known about the distribution of different tick species as well as about prevalence of tick-borne pathogens (TBP) such as *Rickettsia* spp., *Borrelia burgdorferi* (*sensu* *lato*), “*Candidatus* Neoehrlichia mikurensis” (CNM), *Anaplasma phagocytophilum* and *Babesia* spp. in ticks from Bulgaria.

*Rickettsia* spp. are obligate intracellular gram-negative bacteria which may be divided into four groups, i.e. the spotted fever group (SFG), the typhus group, the ancestral group, and the transitional group. Tick-borne rickettsioses are caused by rickettsiae from SFG [1]. Symptoms of spotted fever may include fever, headache, and abdominal pain. The Mediterranean spotted fever (MSF), which is mainly caused by *R. conorii*, may have a far more severe outcome. MSF is endemic in some regions in Bulgaria, and severe cases have been reported [2, 3]. *Ixodes ricinus*, *Dermacentor reticulatus* and *Rhipicephalus* spp. are mainly involved in the circulation of *Rickettsia* species in Europe.

Lyme borreliosis (Lyme disease) is the most common tick-borne disease in Bulgaria [4, 5] where *B. burgdorferi* (*s*.*l*.) was found not only in its main vector *Ixodes ricinus* but additionally in a few *Dermacentor marginatus* and *Haemaphysalis punctata* specimens [6]. There are six known genospecies of *B. burgdorferi* (*s*.*l*.) occuring in Bulgaria, i.e. *B. afzelii*, *B. burgdorferi* (*s*.*l*.), *B. garinii*, *B. lusitaniae*, *B. spielmanii* and *B. valaisiana* [4]. There are only a few studies on *B. burgdorferi* (*s*.*l*.) in ticks from Bulgaria; however, these studies report high prevalence rates (32–40%) [4, 7].

“*Candidatus* Neoehrlichia mikurensis” (CNM) is also a gram-negative, obligate intracellular bacterium transmitted by ticks that are of considerable risk for human and animal health [8–10]. To our knowledge, the occurrence of CNM has not been reported in Bulgaria thus far.

*Anaplasma phagocytophilum* is a gram-negative obligate intracellular bacterium belonging to the family *Anaplasmataceae*. In Europe, *A. phagocytophilum* is mainly transmitted by *I. ricinus*. To our knowledge, only one study from Bulgaria examined *A. phagocytophilum* in *I. ricinus* ticks, with a surprisingly high prevalence (35%) [7].

*Babesia* spp. are single-celled Apicomplexa which parasitize erythrocytes and may cause babesiosis in humans, horses, dogs and cattle. Ticks such as *Rhipicephalus sanguineus*, *I. ricinus* and *D. reticulatus* are the most important vectors for several different *Babesia* species in Bulgaria [11].

To our knowledge, until now, most of the studies examining ticks and tick-borne pathogens in Bulgaria were conducted on small sample sizes mainly from central Bulgaria [4, 5, 12]. The current study is focused on ticks from the largest protected area in Bulgaria, Strandja Nature Park, which is located in the south-eastern part of the country at the Black Sea [13]. It is often frequented by visitors for leisure activities in the natural surroundings and thus of public health relevance.

As knowledge is lacking on the distribution of ticks and tick-borne bacteria and parasites in this area, the aim of this study was to examine the prevalence of tick-borne pathogens in ticks occurring in this region.

## Methods

### PCR-screening for tick-borne bacteria and parasites

cDNA from 1541 ticks collected from the vegetation by flagging method (*n* = 1140), from humans by human-landing catch (*n* = 74) and from hosts (*n* = 327): dogs (*n* = 56), cattle (*n* = 83), tortoises (*n* = 22), goats (*n* = 20), rodents (*n* = 60), shrews (*n* = 1) and hedgehogs (*n* = 85) in the Burgas Province (south-east Bulgaria) was provided by Ohlendorf et al. (unpublished) (Table 1). A description of sampling sites and sample processing will be published elsewhere. Pooled cDNA samples were screened by quantitative real-time PCR (qPCR) for the presence of *Rickettsia* spp. targeting the *gltA* gene (70 bp) [14], *B. burgdorferi* (*s*.*l*.) complex targeting the *p41* gene (96 bp) [15], *A. phagocytophilum* targeting the *msp2* gene (77 bp) [16], and CNM targeting the *groEL* gene (99 bp) [10, 17]. All qPCR reactions were carried out using Mx3000P Real-Time Cycler (Stratagene, Agilent Technologies Deutschland GmbH, Waldbronn, Germany). To detect *Babesia* spp., a conventional PCR targeting the *18S* rRNA gene (411–452 bp) [18] was carried out. This PCR also amplifies DNA of *Theileria* spp. but is only referred to *Babesia* spp. in the following text. All positive pools were further analyzed separately, to identify positive samples, except for *Rickettsia* spp. due to high prevalence. To determine infection levels of *Rickettsia* spp. in ticks, 563 samples were selected (based on established criteria such as collecting method and location, tick species, development stage and sex) for qPCR. Then randomly selected *Rickettsia*-positive samples yielding a cycle threshold (C_t_) value below 35 were further investigated by a conventional PCR targeting 811 bp of the *ompB* (the outer membrane protein B) gene [19]. Samples positive for *B. burgdorferi* (*s*.*l*.) by qPCR (C_t_ < 33) were further examined by single-locus sequence typing targeting the *recG* gene (722 bp) [20, 21]. Conventional PCRs were carried out in the Eppendorf MasterCycler Gradient Thermal Cycler (Eppendorf AG, Hamburg, Germany) and the products were visualized by gel-electrophoresis on 1.5% agarose gel stained with Midori Green (NIPPON, Genetics, Düren, Germany). Positive conventional PCR products, all for *Babesia* spp. and a randomized selection for *Rickettsia* spp. (*n* = 31) and *Borrelia* spp. (*n* = 2), were purified using the NucleoSpin® and PCR Clean-up Kit (Macherey-Nagel, Düren, Germany) according to the manufacturer’s instructions. Purified PCR products were sequenced commercially (Interdisziplinäres Zentrum für Klinische Forschung, Leipzig, Germany) with forward and reverse primers used for PCR. Obtained sequences were assembled and analyzed with Bionumerics (Version 7.6) and compared to GenBank entries in NCBI BLAST.

**Table 1:** Ticks collected in Bulgaria, 2012

Sampling site	Collection method	Total	Developmental stage				Genus				
			Larvae	Nymphs	Adults		*Ixodes*	*Rhipicephalus*	*Hyalomma*	*Haemaphysalis*	*Dermacentor*
					Male	Female					
Silkosiya	F, SM, T, HLC	670	378 (56.4)	266 (39.7)	12 (1.8)	14 (2.1)	658 (98.2)	4 (0.6)	8 (1.2)	0	0
Stoilovo	F, G, D, HLC	282	55 (19.5)	152 (53.9)	37 (13.1)	38 (13.5)	227 (80.5)	44 (15.6)	7 (2.5)	2 (0.7)	2 (0.7)
Sredoka	F, HLC	239	101 (42.2)	125 (52.3)	5 (2.1)	8 (3.3)	238 (99.6)	1 (0.4)	0	0	0
Kosti	F, SM, D, HLC	127	67 (52.7)	51 (40.2)	2 (1.6)	7 (5.5)	88 (69.3)	38 (29.9)	1 (0.8)	0	0
Malko Tarnovo	F, C, HLC	87	0	0	48 (55.2)	39 (44.8)	4 (4.6)	52 (59.8)	31 (35.6)	0	0
Bulgari	F	47	16 (34.1)	30 (63.8)	1 (2.1)	0	45 (95.7)	2 (4.3)	0	0	0
Sinemorets	HLC	16	1 (6.2)	3 (18.8)	8 (50.0)	4 (25.0)	9 (56.0)	7 (43.8)	0	0	0
Zvezdets	F	55	9 (16.4)	39 (70.9)	3 (5.4)	4 (7.3)	55 (100)	0	0	0	0
Ropotamo	D	6	0	1 (16.7)	1 (16.7)	4 (66.6)	3 (50.0)	0	3 (50.0)	0	0
Wildlife Reh. Centre	T	12	0	0	10 (83.3)	2 (16.7)	0	2 (16.7)	10 (83.3)	0	0
Total		1541	627 (40.7)	667 (43.3)	127 (8.2)	120 (7.8)	1327 (86.1)	150 (9.8)	60 (3.9)	2 (0.1)	2 (0.1)

*Abbreviations*: *C* cattle, *D* dogs, *F* flagging, *G* goats, *HLC* human landing catch, *SM* small mammals, *T* tortoises

### Statistical analysis

Confidence intervals (95%CI) for the prevalence in questing and engorged ticks were determined by the Clopper and Pearson method using the GraphPad Software (GraphPad Software Inc., San Diego, Ca., USA). The Fisher’s exact was applied to test the independence of compared prevalence values.

## Results

### PCR results and sequence analysis for tick-borne bacteria and parasites from all ticks

In total, 23.2% of all ticks (358 out of 1541) were positive for at least one of the investigated pathogens (*Rickettsia* spp., *B. burgdorferi* (*s*.*l*.), CNM, *A. phagocytophilum*, or *Babesia* spp.).

Among positive subadult life stages (larvae and nymphs, *n* = 302), the predominant genus was *Ixodes* spp. (99.7%) and only one individual of *Rhipicephalus* spp. (0.3%) was found. Infected adult development stages (females and males, *n* = 56) belonged mostly to *Hyalomma* spp. (50.8%, *n* = 31), followed by *Ixodes* spp. (31.2%, *n* = 19), *Rhipicephalus* spp. (16.4%, *n* = 10) and only one *Dermacentor* spp. (1.6%). The highest prevalence of investigated TBP was detected for *Rickettsia* spp. which was significantly more often detected than any other pathogen (48.3%, *n* = 272, *P* < 0.001, CI: 45.9–54.28%). But *A. phagocytophilum* (6.2%, *n* = 95, *P* < 0.001, CI: 5.06–7.48%) was still significantly more often detected than *B. burgdorferi* (*s*.*l*.) (1.7%, *n* = 26), *Babesia* spp. (0.4%, *n* = 6), and CNM (0.06%, *n* = 1).

*Rickettsia* spp. were found most significantly in *I. ricinus* (66.6%, *n* = 237, *P* < 0.001, CI: 61.52–71.28%), followed by *Hyalomma* spp., *D. marginatus* and *Rhipicephalus* spp. Sequencing of selected samples (*n* = 31) revealed presence of three *Rickettsia* species (Table 2): (i) *R. monacensis* (61.3%, *n* = 19) showing a similarity from 99 to 100% to three different sequences on GenBank (accession nos. KU961543, EU330640, JN036418), followed by (ii) *R. aeschlimannii* (25.8%, *n* = 8) showing 100% identity to a sequence from GenBank (KU961544) and (iii) *R. helvetica* (12.9%, *n* = 4) with 100% identity to a GenBank sequence with accession no. KU310591. All *R. monacensis* and *R. helvetica* sequences were detected in *I. ricinus* samples (from vegetation, dogs and goats), while *R. aeschlimannii* was detected in ticks from dogs and cattle: *Hy. anatolicum* (*n* = 1), *Hy. excavatum* (*n* = 2), *Hy. marginatum* (*n* = 4), and *Rhipicephalus* spp. (*n* = 1). *Borrelia burgdorferi* (*s*.*l*.) was detected only in *I. ricinus* (1.9%, *n* = 25) and *Ixodes* spp. (2.8%, *n* = 1). Sequenced *Borrelia* samples (*n* = 2) belonging to *B. afzelii* (100% identity with the sequence with the GenBank accession number CP009058) were detected in one *I. ricinus* tick collected from vegetation and one from a hedgehog. CNM was detected only in one specimen of tested ticks (0.1%, *n* = 1) which was identified as *Ixodes ricinus* and collected from vegetation. For *A. phagocytophilum* the prevalence was significantly higher in *Ixodes* spp. (38.9%, *n* = 14, *P* < 0.001, CI: 24.75–55.17%), than in any other genus. DNA of *Babesia* spp. was found in 0.4% (*n* = 6) of investigates ticks and all of them were collected from hosts. *Babesia* spp. was detected in *Hyalomma* spp. (100%, *n* = 1), *Hy. marginatum* (3.3%, *n* = 1), *R. bursa* (3.2%, *n* = 3) and *I. ricinus* (0.06%, *n* = 1). There were two *Babesia* and one *Theileria* species found in ticks from the current study: (i) *B. microti* was detected in *I. ricinus* from *Apodemus flavicollis*, the yellow-necked mouse (92% identity with KX591647); (ii) *B. caballi* in *Hy. marginatum* from cattle (100% identity with KX375824) and (iii) *T. buffeli* detected in *R. bursa* from cattle (showing 100% identity with KX375823). *Theileria buffeli* was also detected in two *Hyalomma* spp. (showing 100% identity with KX375822), also from cattle. All ticks infested with *Babesia* spp. were also infected with other pathogens. Co-infections (Table 3) were detected in 2.5% (*n* = 39) of tested tick specimens, mainly in *Ixodes* spp.

**Table 2:** Sequencing results of tested samples from Bulgaria, 2012 in comparison to GenBank entries from NCBI

Tick species	Life stage				Sampling method	Pathogen				% of the highest identity^a^	GenBank ID	Reference
	L	N	Adults			*Rickettsia*	*Borrelia*	*Babesia*	Sequenced species			
			M	F								
*I. ricinus*	×				small mammals			×	*B. microti*	92	KX591647	[61]
*Hy. marginatum*				×	cattle			×	*B. caballi*	100	KX375824	unpublished
*R. bursa*				×	cattle			×	*T. buffeli*	100	KX375823	[66]
*R. bursa*				×	cattle			×	*T. buffeli*	100	KX375822	[66]
*R. bursa*				×	cattle			×	*T. buffeli*	100	KX375822	[66]
*Hyalomma* spp*.*				×	cattle			×	*T. buffeli*	100	KX375822	[66]
*I. ricinus*		×			flagging		×		*Bo. afzelii*	100	CP009058	[72]
*I. ricinus*		×			small mammals		×		*Bo. afzelii*	100	CP009058	[72]
*Hy. anatolicum*			×		dogs	×			*R. aeschlimannii*	100	KU961544	unpublished
*Hy. marginatum*				×	cattle	×			*R. aeschlimannii*	100	KU961544	unpublished
*Rhipicephalus* spp*.*		×			dogs	×			*R. aeschlimannii*	100	KU961544	unpublished
*Hy. marginatum*			×		cattle	×			*R. aeschlimannii*	100	KU961544	unpublished
*Hy. marginatum*			×		cattle	×			*R. aeschlimannii*	100	KU961544	unpublished
*Hy. marginatum*			×		cattle	×			*R. aeschlimannii*	99	KU961544	unpublished
*Hy. excavatum*				×	cattle	×			*R. aeschlimannii*	99	KU961544	unpublished
*Hy. excavatum*				×	dogs	×			*R. aeschlimannii*	100	KU961544	unpublished
*I. ricinus*	×				flagging	×			*R. helvetica*	100	KU310591	unpublished
*I. ricinus*	×				flagging	×			*R. helvetica*	100	KU310591	unpublished
*I. ricinus*	×				flagging	×			*R. helvetica*	100	KU310591	unpublished
*I. ricinus*	×				flagging	×			*R. helvetica*	99	KU310591	unpublished
*I. ricinus*				×	dogs	×			*R. monacensis*	100	KU961543	unpublished
*I. ricinus*				×	dogs	×			*R. monacensis*	100	KU961543	unpublished
*I. ricinus*				×	goats	×			*R. monacensis*	100	KU961543	unpublished
*I. ricinus*		×			flagging	×			*R. monacensis*	99	KU961543	unpublished
*I. ricinus*		×			flagging	×			*R. monacensis*	100	KU961543	unpublished
*I. ricinus*		×			flagging	×			*R. monacensis*	100	KU961543	unpublished
*I. ricinus*		×			flagging	×			*R. monacensis*	99	KU961543	unpublished
*I. ricinus*		×			flagging	×			*R. monacensis*	100	KU961543	unpublished
*I. ricinus*		×			flagging	×			*R. monacensis*	99	KU961543	unpublished
*I. ricinus*		×			small mammals	×			*R. monacensis*	100	KU961543	unpublished
*I. ricinus*	×				flagging	×			*R. monacensis*	100	EU330640	unpublished
*I. ricinus*	×				flagging	×			*R. monacensis*	100	KU961543	unpublished
*I. ricinus*	×				flagging	×			*R. monacensis*	100	EU330640	unpublished
*I. ricinus*	×				flagging	×			*R. monacensis*	100	JN036418	[45]
*I. ricinus*	×				flagging	×			*R. monacensis*	100	KU961543	unpublished
*I. ricinus*	×				flagging	×			*R. monacensis*	100	KU961543	unpublished
*I. ricinus*	×				flagging	×			*R. monacensis*	100	JN036418	[45]
*I. ricinus*	×				flagging	×			*R. monacensis*	100	KU961543	unpublished
*I. ricinus*	×				flagging	×			*R. monacensis*	100	KU961543	unpublished

^a^Percentage identity to sequences deposited in the GenBank database

*Abbreviations*: *L* larvae, *N* nymphs, *M* males, *F* females

**Table 3:** Number of co-infections with *Anaplasma phagocytophilum*, *Rickettsia* spp., *Borrelia* spp. and *Babesia* spp. in tick genera collected in Bulgaria, 2012

Genus	Pathogens					
	*Rickettsia + Anaplasma + Babesia*	*Rickettsia + Anaplasma*	*Rickettsia + Borrelia*	*Anaplasma + Borrelia*	*Rickettsia + Babesia*	*Rickettsia + Anaplasma + Borrelia*
*Ixodes*	–	19	1	12	1	1
*Rhipicephalus*	1	–	–	–	2	–
*Hyalomma*	1	–	–	–	1	–
Total	2	19	1	12	4	1

### Prevalence of tick-borne bacteria and parasites in ticks collected only from vegetation

Ticks collected from vegetation (*n* = 1214) were positive for four of the five investigated pathogens (Table 4), *Rickettsia* spp. (59.12%; *n* = 214), *A. phagocytophilum* (2.47%; *n* = 30), *B. burgdorferi* (*s.l*.) (0.91%; *n* = 11) and CNM (0.08%; *n* = 1) which was detected only in ticks from vegetation. No *Babesia* spp. infections were detected. The highest diversity of TBP was found among *Ixodes* spp. (four pathogens). Ticks from vegetation positive for *Rickettsia* spp., CNM and *Borrelia burgdorferi* (*s*.*l*.) were exclusively belonging to the genus *Ixodes*. Moreover, CNM was found only in one tick from vegetation. Ticks positive for *A. phagocytophilum* were belonging to the genera *Ixodes* and *Rhipicephalus*.

**Table 4:** Prevalence of tick-borne pathogens in tick species collected from vegetation and HLC in Bulgaria, 2012

Species	Number of ticks					Number of positive/tested ticks (%) [95% CI]			
	L	N	M	F	Total	*Rickettsia* spp.	*Anaplasma phagocytophilum*	*Borrelia burgdorferi*	“*Ca*. N. mikurensis”
*Ixodes* spp.	6	1	0	0	7	–	0/7 (0)	0/7 (0)	0/7 (0)
*I. ricinus*	534	579	23	26	1162	214/340 (62.94) [57.69–67.91]	29/1162 (2.5) [1.73–3.57]	11/1162 (0.95) [0.51–1.71]	1/1162 (0.09) [0–0.54]
*Hy. marginatum*	0	0	5	2	7	0/1 (0)	0/7 (0)	0/7 (0)	0/7 (0)
*Hy. anatolicum*	0	0	1	0	1		0/1 (0)	0/1 (0)	0/1 (0)
*Hy. excavatum*	0	0	0	3	3		0/3 (0)	0/3 (0)	0/3 (0)
*Rhipicephalus* spp.	1	0	0	0	1	0/21(0)	0/1(0)	0/1 (0)	0/1 (0)
*R. bursa*	21	0	3	0	24		0/24 (0)	0/24 (0)	0/24 (0)
*R. sanguineus*	0	0	2	3	5		1/5 (20) [2.03–64.04]	0/5 (0)	0/5 (0)
*R. rossicus*	0	0	1	0	1		0/1 (0)	0/1 (0)	0/1 (0)
*R. turanicus*	0	0	0	1	1		0/1 (0)	0/1 (0)	0/1 (0)
*Haemaphysalis punctata*	0	1	0	1	2	–	0/2 (0)	0/2 (0)	0/2 (0)
Total	562	581	35	36	1214	214/362 (59.12) [53.98–64.06]	30/1214 (2.47) [1.72–3.52]	11/1214 (0.91) [0.49–1.64)]	1/1214 (0.08) [0–0.51]

*Abbreviations*: *CI* confidence interval, *F* females, *L* larvae, *N* nymphs, *M* males. All tested samples were *Babesia*-negative

### Prevalence of tick-borne bacteria and parasites in ticks collected only from hosts

Ticks collected from hosts (*n* = 327) were infected by four out of the five investigated pathogens (Table 5), *A. phagocytophilum* (19.88%, *n* = 65), *Rickettsia* spp. (28.86%, *n* = 58), *B. burgdorferi* (*s*.*l*.) (4.59%, *n* = 15), and *Babesia* spp. (1.83%, *n* = 6) which was found only in ticks from hosts. CNM was not detected in ticks from hosts. The highest diversity of TBP was found among *Ixodes* spp. (four pathogens) and the lowest among *Dermacentor* spp. (one pathogen). *Rickettsia* spp. was found in all tick genera collected from hosts (*Hyalomma*, *Ixodes*, *Rhipicephalus* and *Dermacentor*). The highest prevalence was detected in *Ixodes*, followed by *Hyalomma*, *Dermacentor* and *Rhipicephalus.* A significantly higher prevalence for *Borrelia* spp. was found in ticks from small mammals (10.3%, *n* = 15, *P* < 0.001, CI: 9.8–30.04%) compared to any other host species.

**Table 5:** Prevalence of tick-borne pathogens in tick species collected from hosts in Bulgaria, 2012

Species	Number of ticks					Number of positive/tested ticks (%) [95% CI]			
	L	N	M	F	Total	*Rickettsia* spp.	*Anaplasma phagocytophilum*	*Borrelia burgdorferi*	*Babesia* spp.
*Ixodes* spp.	12	16	0	1	29	–	14/29 (48.28) [31.39–65.57]	1/29 (3.45) [0–18.63]	0/29 (0)
*I. ricinus*	52	55	3	19	129	23/36 (63.89) [47.52–77.58]	47/129 (36.43) [28.62–45.03]	14/129 (10.85) [6.46–17.51]	1/129 (0.78) [0–4.69]
*Hyalomma* spp.	0	0	0	1	1	27/49 (55.1) [41.31–68.12]	1/1 (100) [16.75–100]	0/1 (0)	1/1 (100) [16.75–100]
*Hy. marginatum*	0	0	21	2	23		0/16 (0)	0/16 (0)	1/16 (6.25) [0–30.31]
*Hy. aegypticum*	0	0	17	1	18		0/18 (0)	0/18 (0)	0/18 (0)
*Hy. anatolicum*	0	0	2	0	2		0/2 (0)	0/2 (0)	0/2 (0)
*Hy. excavatum*	0	0	0	4	4		0/4 (0)	0/4 (0)	0/4 (0)
*Hy. scupense*	0	0	0	1	1		0/1 (0)	0/1 (0)	0/1 (0)
*Dermacentor marginatus*	0	0	0	2	2	1/2 (50.00) [9.45–90.55]	0/2 (0)	0/2 (0)	0/2 (0)
*Rhipicephalus* spp.	1	12	0	1	14	7/114 (6.14) [2.79–12.35]	0/14 (0)	0/14 (0)	0/14 (0)
*R. bursa*	0	0	31	38	69		3/69 (4.35) [0.99–12.52]	0/69 (0)	3/69 (4.35) [0.99–12.52]
*R. sanguineus*	0	3	16	13	32		0/32 (0)	0/32 (0)	0/32 (0)
*R. rossicus*	0	0	1	1	2		0/2 (0)	0/2 (0)	0/2 (0)
*R. turanicus*	0	0	0	1	1		0/1 (0)	0/1 (0)	0/1 (0)
Total	65	86	91	85	327	58/201 (28.86) [23.02–35.48]	65/327 (19.88) [15.9–24.56]	15/327 (4.59) [2.74–7.49]	6/327 (1.83) [0.75–4.04]

*Abbreviations*: *CI* confidence interval, *F* females, *L* larvae, *N* nymphs, *M* males

All tested samples were CNM-negative

The prevalence for *A. phagocytophilum* was significantly higher in ticks from hosts in comparison to ticks from vegetation (19%, *n* = 65, *P <* 0.001, CI: 15.9–24.56%). All *Anaplasma*-positive ticks from hosts belonged to all investigated genera except for *Dermacentor* spp. The prevalence for *B. burgdorferi* (*s*.*l*.) was significantly higher in ticks from hosts than from vegetation (4.6%, *n* = 15, *P <* 0.001, CI: 6.2–16.36%).

*Babesia* spp. DNA was detected only in ticks from hosts and was significantly more often detected in ticks from one location, Malko Tarnovo (5.75%, *n* = 5, *P* < 0.001, CI: 2.16–13.07%), where most ticks were collected from cattle.

## Discussion

Until today, studies in Bulgaria were mostly focused on Lyme disease in humans, sheep, cows and dogs [4, 22, 23]. Most studies from Bulgaria on tick-borne pathogens are serological surveys in humans, cattle and dogs [2, 22–24] and there are only a few studies investigating ticks for tick-borne pathogens [5, 22, 25]. Further, these studies examined only a small sample size of ticks (*n* = 94–299) [4, 6, 7, 12]. The current study reports tick-borne bacteria and parasites on a larger scale in a nature park at the Black Sea in Bulgaria with a high frequency of visitors.

*Ixodes ricinus* was the predominant tick species in this study which is not surprising as it is the most common tick species in the Northern Hemisphere [26]. The infection rate for tick-borne pathogens was also significantly higher in *I. ricinus* compared to all other tick species, which is not unusual as *I. ricinus* is known to be the most important vector of tick-borne pathogens in Europe [27].

*Rickettsia* spp. were found in every tick genus examined. However, a higher diversity of tick species infected by *Rickettsia* spp. were collected from hosts (ticks belonging to *Ixodes*, *Hyalomma*, *Dermacentor* and *Rhipicephalus*) than from vegetation (only *Ixodes*). In general, the prevalence in questing ticks was higher compared to the one obtained from ticks collected from animals. The infection levels in almost all tick genera (*Ixodes* - both from vegetation and hosts, *Hyalomma* and *Dermacentor* from hosts) were very high, i.e. at least 50%, except for *Rhipicephalus* ticks from hosts which were infected only in few percentage. Interestingly, most *Rickettsia*-positive ticks collected from small mammals, were parasitizing southern white-breasted hedgehogs, *Erinaceus concolor*. There are no data about *Rickettsia* infection in ticks collected from *E. concolor* but other hedgehog species such as *E. europaeus,* are known to serve as potential reservoirs for certain *Rickettsia* spp. from urban and suburban areas [28–30]. Sequence analysis revealed a variety of different *Rickettsia* species such as *R. helvetica*, *R. aeschlimannii* and *R. monacensis* in the current study. All of them are considered as agents of human diseases and occur in Europe [1, 31]. *Rickettsia* species were detected only in their respective vectors: *R. helvetica* and *R. monacensis* were exclusively in *I. ricinus*, and *R. aeschlimannii* was found only in *Hyalomma* spp. [1, 32]. All *R. aeschlimannii* samples were very closely associated with the Crimean isolate obtained from *Hy. marginatum* (KU961544, unpublished). Migrating birds from Africa are considered as reservoirs for *R. aeschlimannii* in Europe and *Hyalomma* spp. are remarkably contributing to its transmission in southern Europe [32, 33]. The *R. helvetica* sequences detected in the current study were almost identical with the one previously detected in *I. persulcatus* from Novosibirsk Region, Russia (KU310591, unpublished). The ubiquitously occurring *R. helvetica* is mostly transmitted by *I. ricinus* ticks which are considered as its main vector and reservoir, but it was previously detected also in tissues of many vertebrates, e.g. rodents, hedgehogs, dogs, deer, birds and dogs [1, 34–36]. *Rickettsia monacensis* sequences obtained in this study had a high similarity to (i) a Crimean isolate acquired from *Ha. punctata* (KU961543, unpublished), (ii) a variant isolated from *I. ricinus* ticks from Germany (EU330640, unpublished), and (iii) a strain detected in *I. ricinus* from an urban park in Munich, Germany (JN036418.1; [37]). Widely distributed in Europe, *R. monacensis* was detected previously not only in *I. ricinus* ticks but on hosts, mainly migratory birds and lizards [38–41]. In the current study, *R. monacensis* was detected in *Ixodes* ticks collected from the southern white-breasted hedgehogs, *Erinaceus concolor* for the first time.

*Borrelia burgdorferi* (*s*.*l*.) was found with a low prevalence (1.7%) compared to other studies (32–37.3%) from Bulgaria [4, 12]. All positive ticks from this study belonged to the genus *Ixodes*, which is in line with previous studies from Bulgaria. However, there is also a study reporting *Borrelia*-positive *D. marginatus* and *Ha. punctata* which were collected from humans with Lyme disease in Bulgaria [6]. In this study, most *Borrelia*-positive ticks were collected from small mammals, especially from *E. concolor*. Sequencing unveiled presence of pathogenic *B. afzelii* with a 100% identity with a sequence obtained from human skin in Austria (CP009058; [42]). Again, there is no information about *Borrelia*-infected ticks collected from *E. concolor*; however, many studies report the prevalence of *Borrelia* species, including *B. afzelii*, in ticks collected from other hedgehog species in the neighbouring country Romania [30, 43, 44].

In this study, CNM was found in a single specimen of *I. ricinus* from vegetation only. To our knowledge, this is the first detection of CNM in Bulgaria. Nevertheless, the prevalence (0.1%) for CNM in this study was lower compared to other studies from central Europe (2.2–45%) [10, 17, 45]. However, results from south-eastern Europe show a similar low prevalence (0–1.3%) leading to the assumption that CNM in ticks is occurring more often in central Europe, where also clinical cases of neoehrlichiosis were reported than in south-eastern Europe where clinical cases are thus far absent [46, 47].

The majority of *Anaplasma phagocytophilum*-positive ticks in this study belonged to *I. ricinus* (over 90%), which is in line with other studies from Europe suggesting *I. ricinus* as the main vector [48, 49]. The current study reports a high prevalence of *A. phagocytophilum* in ticks collected from small mammals compared to questing ticks and ticks collected from any other animal species. This finding is in contrast to other European studies reporting low or even zero prevalence in ticks collected from small mammal species such as *Apodemus* spp. and *Myodes* spp. [45, 50]. However, one should take into account that infected ticks obtained from small mammals in this study were collected mainly from southern white-breasted hedgehogs, *E. concolor*. There are no available data on *Anaplasma* infections in ticks from *E. concolor* but in general, the hedgehog *E. europaeus* is a suspected reservoir host for *A. phagocytophilum* [30, 43, 51, 52]. In Romania which is a neighbouring country to Bulgaria, *A. phagocytophilum* was detected in ticks collected from another hedgehog species, *Erinaceus roumanicus* with a prevalence of 12% [44].

*Babesia* spp. and *Theileria* spp. were found with a remarkable low prevalence in ticks in this study (less than 1%) in comparison to the prevalence in blood samples of dogs and ticks collected from humans and the environment from Bulgaria in previous studies (3.6–31.4%) [11, 24]. *Babesia* spp. and *Theileria* spp. were detected only in ticks collected from hosts and were belonging to three genera: *Hyalomma*, *Rhipicephalus* and *Ixodes*, which is not surprising as these tick species are known to be vectors for these protozoans especially in neighbouring countries such as Turkey [53–55]. Sequence analysis revealed the presence of three species. *Babesia microti* detected in *I. ricinus* from the yellow-necked mouse *A. flavicollis*,, which is known to serve as a reservoir, was most closely related with an isolate obtained from questing *I. ricinus* in Kyiv Botanical Garden, Ukraine (KX591647; [56]). *Babesia microti* is responsible for human babesiosis cases mostly in the USA, but it was also detected in *I. ricinus* ticks in Europe [57, 58]. However, European strains of *B. microti* are known to be less pathogenic. Only the ‘Jena’ strain is considered as pathogenic for humans in Europe [57]. The sequences for *B. caballi* detected in a female *Hy. marginatum* tick feeding on cattle in the current study, showed the closest similarity to a sequence found as well in a female *Hy. marginatum* tick collected from vegetation in Italy (KX375824, unpublished). *Babesia caballi* is known as the etiological agent of equine piroplasmosis, and ticks from following genera have been identified as significant vectors of this protozoon: *Boophilus*, *Dermacentor*, *Haemaphysalis*, *Hyalomma* and *Rhipicephalus* [59]. *Theileria buffeli* detected in *R. bursa*, and *Hyalomma* spp. from cattle in the current study was identical with two sequences obtained from *R. annulatus* nymphs parasitizing cattle in Italy which were most likely misnamed as *T. sergenti* (KX375822, KX375823; [60]). According to Uilenberg [61], there is confusion in the nomenclature, and *T. sergenti* should be named as *T. buffeli* which is responsible for bovine theileriosis worldwide, since the name ‘*T. sergenti*’ has been used earlier to describe a *Theileria* species infesting sheep [62, 63].

Altogether, the prevalence in ticks from hosts was higher for most pathogens. Moreover, more tick genera collected from hosts were found to be positive in general in comparison to ticks which were collected from vegetation. These facts point out that the uptake of pathogens during a blood meal on potential reservoir hosts is more probable than the vertical transmission of the pathogen in ticks. Co-infections in ticks were detected in combination with almost all pathogens besides CNM and the combination of infection of *Borrelia* spp. and *Babesia* spp. Co-infections have been described for *Rickettsia* spp., *Borrelia* spp., *Babesia* spp. and *A. phagocytophilum* [45, 64]. As co-infection levels in this study were rather low, no significant combination of pathogens could be found.

## Conclusions

In conclusion, this study presents the prevalence of tick-borne bacteria and parasites in ticks on a large scale for the first time in a natural reserve in Bulgaria. To our knowledge, this study reports the first detection of “*Candidatus* Neoehrlichia mikurensis” and *R. aeschlimannii* in ticks from Bulgaria. A high diversity of tick-borne pathogens (*R. monacensis, A. phagocytophilum* and *B. afzelii*) was detected in ticks collected from the southern white-breasted hedgehog, *E. concolor*, for the first time suggesting it as a host maintaining circulation of tick-borne pathogens. Although most tick-borne pathogens studied were only found with a low prevalence, the prevalence of *Rickettsia* spp. was very high and diverse species were found. This may be of health impact as humans may suffer from spotted fever after having a tick bite from this region in Bulgaria.

## Abbreviations

cDNA: complementary DNA
CI: confidence interval
CNM: *"Candidatus* Neoehrlichia mikurensis"
HLC: human-landing catch
MSF: Mediterranean spotted fever
PCR: polymerase chain reaction
qPCR: quantitative real-time polymerase chain reaction
RNA: ribonucleic acid
SFG: spotted fever group
TBD: tick-borne disease
TBP: tick-borne pathogens
